# miRNA Regulons Associated with Synaptic Function

**DOI:** 10.1371/journal.pone.0046189

**Published:** 2012-10-08

**Authors:** Maria Paschou, Maria D. Paraskevopoulou, Ioannis S. Vlachos, Pelagia Koukouraki, Artemis G. Hatzigeorgiou, Epaminondas Doxakis

**Affiliations:** 1 Basic Neurosciences Division, Biomedical Research Foundation of the Academy of Athens, Athens, Greece; 2 Institute of Molecular Oncology, Biomedical Sciences Research Center “Alexander Fleming” Vari, Greece; 3 Department of Computer and Communication Engineering, University of Thessaly, Volos, Greece; University of Illinois-Chicago, United States of America

## Abstract

Differential RNA localization and local protein synthesis regulate synapse function and plasticity in neurons. MicroRNAs are a conserved class of regulatory RNAs that control mRNA stability and translation in tissues. They are abundant in the brain but the extent into which they are involved in synaptic mRNA regulation is poorly known. Herein, a computational analysis of the coding and 3′UTR regions of 242 presynaptic and 304 postsynaptic proteins revealed that 91% of them are predicted to be microRNA targets. Analysis of the longest 3′UTR isoform of synaptic transcripts showed that presynaptic mRNAs have significantly longer 3′UTR than control and postsynaptic mRNAs. In contrast, the shortest 3′UTR isoform of postsynaptic mRNAs is significantly shorter than control and presynaptic mRNAs, indicating they avert microRNA regulation under specific conditions. Examination of microRNA binding site density of synaptic 3′UTRs revealed that they are twice as dense as the rest of protein-coding transcripts and that approximately 50% of synaptic transcripts are predicted to have more than five different microRNA sites. An interaction map exploring the association of microRNAs and their targets revealed that a small set of ten microRNAs is predicted to regulate 77% and 80% of presynaptic and postsynaptic transcripts, respectively. Intriguingly, many of these microRNAs have yet to be identified outside primate mammals, implicating them in cognition differences observed between high-level primates and non-primate mammals. Importantly, the identified miRNAs have been previously associated with psychotic disorders that are characterized by neural circuitry dysfunction, such as schizophrenia. Finally, molecular dissection of their KEGG pathways showed enrichment for neuronal and synaptic processes. Adding on current knowledge, this investigation revealed the extent of miRNA regulation at the synapse and predicted critical microRNAs that would aid future research on the control of neuronal plasticity and etiology of psychiatric diseases.

## Introduction

The synapse is a highly regulated specialized asymmetric structure comprised of a presynaptic terminal having the molecular machinery for neurotransmitter release and a postsynaptic compartment containing the proteins required for neurotransmitter uptake and signal transduction. Synaptic contact is maintained through structural and functional coupling of a repertoire of proteins in both of these compartments [1]. Many of the proteins that are present in synapses are transported to terminals on kinesin motors particularly during the initiation phase of synapse formation, while a great number of other proteins are locally translated during differentiation and maturation [2], [3]. In the later case, the asymmetric localization of mRNAs is used to limit protein expression to these distinct compartments of the cell. Stimulus-induced remodeling of synaptic strength, also known as synaptic plasticity, occurs at synaptic terminal, in part, as a result of rapid translation of these localized mRNAs. Consequently, dynamic regulatory mechanisms for both quantitative and qualitative translation of these mRNAs are required. These mechanisms are currently under intense investigation and may involve RNA binding regulators such as RNA binding proteins and microRNAs (miRNAs) [4], [5].

The discovery of microRNAs has revealed an additional layer of gene regulation during organismal development [6], [7], [8]. miRNAs are approximately 22-nucleotide in length endogenous non-coding, double-stranded RNA molecules that base pair to complementary sequences on the 3′ un-translated region (3′UTR) of mRNAs repressing their translation. Each miRNA is estimated to regulate multiple functionally-related target mRNAs, and the combinatorial action of miRNAs is expected to regulate the expression of hundreds of mRNAs [9], [10], [11]. Currently, over 1500 miRNAs have been identified in humans (miRBase 18) [12]. They have a wide variety of expression patterns, and many are differentially expressed during development or disease [13], [14]. More recently, together with components of the inhibitory miRNA-induced silencing complex, they have been identified in postsynaptic densities, indicating that their action maybe restricted, under specific conditions, to discrete loci within cells [15], [16]. In addition, emerging evidence suggests that miRNA turnover is linked to neuronal activity [17].

With the advent of computational algorithms and tools to predict miRNA-mRNA target interactions there has been a flourish in our understanding of miRNA function. Although computational analysis suffers from drawbacks such as high signal to noise ratio, the high speed of prediction and its prowess of analyzing large data sets, free from interference, make it an ideal tool for initial screenings. Alongside, being skill-intensive and time consuming, the experimental methodologies are marred by difficulties arising from indirect target effects, tissue and age specificity, interference from intracellular structures (P-bodies, stress granules) and multiple levels of gene expression control.

A comprehensive analysis of miRNA and synaptic mRNA interactions has not been reported and we know little of miRNA impact at the synapse. The large number of different miRNAs in the brain coupled with their high and differential degree of expression suggests they may facilitate refined integration and concert of action at the soma and synapses. Here, it was predicted that the great majority of synaptic proteins is miRNA targets and identified a small set of miRNAs that could potentially influence global synaptic protein levels. These findings should aid research efforts to narrow down the list of relevant miRNAs for subsequent experimental analysis into understanding synaptic function and the etiology of psychiatric and neurodegenerative diseases.

## Methods

**Figure 1:**
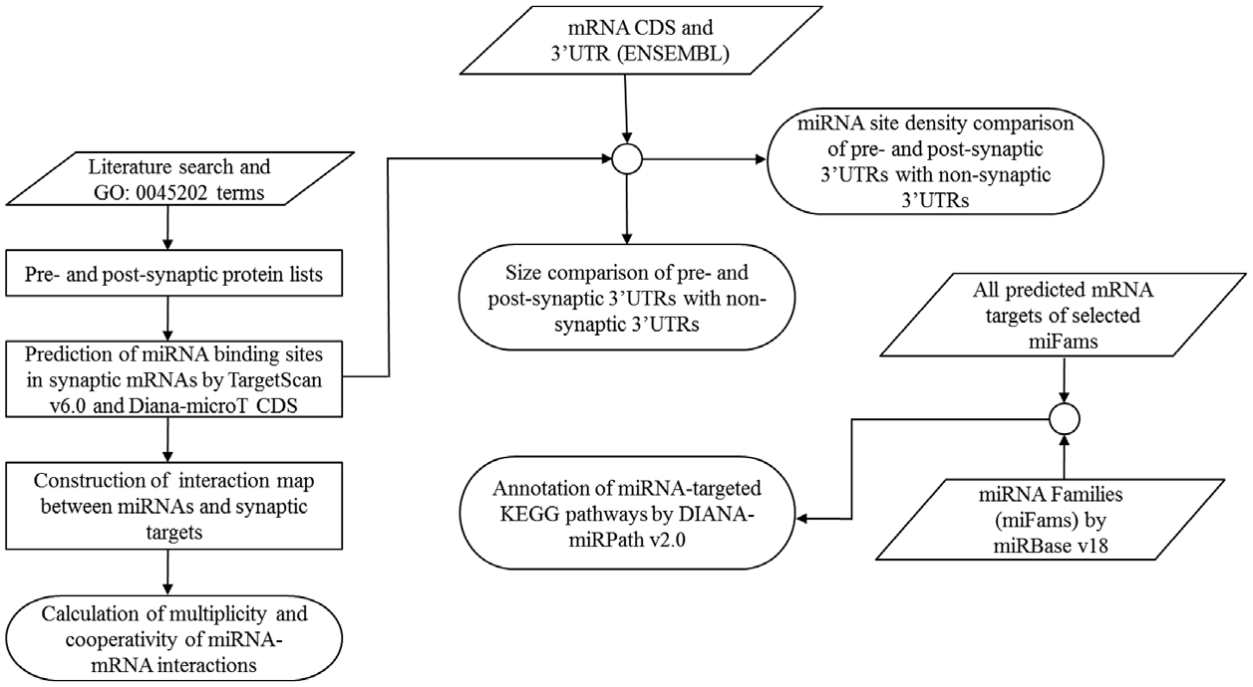
Schematic representation of the workflow.

In current study, we determined whether pre- and post- synaptic proteins are likely targets of miRNA regulation and provided analysis of these interactions. Figure 1 represents a stepwise workflow of this study.

### Selection of input genes

A wide spectrum of proteins participates at the different stages of synapse development. They include proteins that are scaffold to the synapse (e.g. RIMs, BSN, PCLO), adhesion molecules (e.g. CDH2, NLGN1, CHL1), components of synaptic vesicle exo/endo-cytosis (e.g. VAMP1, SNAP25, AMPH) and postsynaptic receptors (GRIN1, GRIK1, CHRNA4). For the purpose of this analysis, an unbiased list of 246 and 306 pre- and post- synaptic proteins was curated from an extensive literature review into synaptic assembly and function [1], [18], [19], [20], [21], [22], [23], [24], [25], [26], [27], [28]. The lists of proteins identified were, then, further enriched with proteins from Gene Ontology (GO), GO: 0045202 synapse subcategories after curating each against available pubmed literature. We have found that a significant number of GO proteins were misplaced in pre- or post- synaptic compartments or both as well as falsely included in the Synapse category likely due to the bioinformatics nature of the database. Nevertheless, we did include in our analysis any protein that appeared to have had even loose connection to synaptic function and could not be excluded.

Five proteins (presynaptic EFNA2, DOC2B, SCAMP1 and postsynaptic GABRR3, GRIP2) were not found in Ensembl 65 database and, thereafter, excluded from further analysis. The full list of proteins, hyperlinked to GeneCards is provided in Table S1 [29].

### 3′UTR sequence extraction of mRNAs and length analysis

Both the longest and shortest 3′UTRs of all 19,346 human mRNA transcripts were retrieved from BioMart (Ensembl 65) [30]. Analyzed data are presented as median (interquartile range). The normality of the distributions was assessed with Kolmogorov-Smirnov test and graphical methods. Kruskal-Wallis test was used for non-parametric multiple group comparisons since the distributions did not follow a Gaussian pattern. Pairwise Mann-Whitney's U test was performed as non-parametric post-hoc test and Benjamini-Hochberg's False Discovery Rate was utilized to detect significant differences and to maintain a family-wise α = 0.05.

### Prediction of common miRNA targets between different algorithms

A large number of computational tools are available for animal miRNA target identification. Two of these, TargetScan v6.0 [31] and DIANA-microT-CDS [32] are extensively used and have been proven robust in analyses against experimentally validated targets of 3′ UTR and more, recently, coding region (CDS) [33], [34]. TargetScan, currently, does not provide online results for target predictions in the CDS of genes and for this the source code was downloaded from www.targetscan.org and used in the analysis. Both algorithms were incorporated in this study's pipeline and were applied on Ensembl 65 mRNA transcripts against all available miRNAs of the latest miRBase 18. Target prediction results were, then, grouped based on the 474 miRNA Families (miFams) classified by miRBase 18. A strict threshold on the binding quality scores of both algorithms was applied. DIANA-microT-CDS predictions were filtered with a transcript-miRNA interaction score of 0.85 while for TargetScan predictions the context+score threshold of −0.4 was utilized. TargetScan total context score was computed as the sum of the context-scores (site-type, 3′ pairing, local AU, target site abundance, seed-pairing and stability) contributing into the specified interaction [31]. The two algorithms predicted more than 4,000 and 5,000 miRNA-gene interactions for the pre- and post- synaptic transcripts, respectively. A voting algorithm was implemented to accept a predicted interaction, only, if both algorithms identified it.

### ClueGo analyses

ClueGo [35] was used to relate pre- and post- synaptic proteins into Gene Ontology biological processes [36]. ClueGO visualized data in a functionally grouped annotation network that reflected the relationships between the terms based on the similarity of their associated genes. The size of the nodes reflected the statistical significance of the terms. The degree of connectivity between terms (edges) was calculated using kappa statistics. The calculated kappa score was, also, used for defining functional groups. The network was, then, laid out using the Organic layout algorithm supported by Cytoscape 2.8.3 [37]. A two-sided hypergeometric test yielded the enrichment for GO terms. Benjamini-Hochberg [38] correction for multiple testing controlled the *P*-values.

### Interaction Analysis

A script was implemented to identify the minimum set of miFams controlling all predicted miRNA-regulated pre- and post- synaptic targets. Following the identification of these sets, specific subgroup combinations were examined to pinpoint those miFams that contributed most miRNA targets. Two groups of ten miFams were finally identified covering a significant portion of the pre- and post- synaptic transcripts. These miFams versus their transcript targets were, then, visualized using Cytoscape 2.8.3.

### Pathway Analysis

The second version of DIANA-miRPath [33] has the capacity to analyze the combinatorial effect of different miRNAs on Kyoto Encyclopedia of Genes and Genomes (KEGG) pathways. Here, it was employed to predict the enriched KEGG pathways of complete miFams. The target prediction threshold was set at 0.85 like it was for the DIANA-microT-CDS miRNA target predictions. Benjamini-Hochberg [38] correction for multiple testing controlled the *P*-values.

## Results

Bioinformatics approaches to analyze transcript regulation by miRNAs offers noteworthy benefits that include a) rapid identification of all miRNAs that are predicted to interact with an mRNA, b) identification of only direct interactions, and c) compilation of informative interaction networks between the miRNAs and corresponding targets. This type of methodology can be exploited to predict molecular hallmarks of distinct biological processes and understand disease pathways. Here, it was employed to explore the post-transcriptional regulation landscape of synaptic proteins with the purpose of narrowing down the list of relevant miRNAs required for subsequent experimentally analysis into synaptic function.

### Characterization of synaptic proteins

For this study, 242 and 304 transcripts previously identified at pre- and post- synaptic terminals were analyzed, respectively (Table S1). To confirm they reflected adequate representation of different pre- and post- synaptic assemblies and functions, the molecular and physiological characteristics were, at start, determined by ClueGo analysis of Gene Ontology terms. The analysis of presynaptic proteins revealed enrichment for presynaptic processes that included ‘axonogenesis’ (32 proteins, p<4.2×10^−9^), ‘focal adhesion assembly’ (6 proteins, p<3.2×10^−3^), ‘ATP hydrolysis coupled proton transport’ (15 proteins, p<9.0×10^−18^) ‘regulation of exocytosis’ (23 proteins, p<2.3×10^−21^), and ‘synaptic vesicle endocytosis’ (9 proteins, p<3.8×10^−10^) (Figure S1, Table S2). The analysis of postsynaptic proteins revealed enrichment for postsynaptic processes that included ‘dendritic spine development’ (12 proteins, 3.5×10^−11^), ‘regulation of postsynaptic membrane potential’ (21 proteins, 4.2×10^−23^), ‘regulation of synapse organization’ (13 proteins, 3.3×10^−10^), and ‘calcium ion transport’ (25 proteins, 1.2×10^−9^) (Figure S2, Table S3).

**Figure 2:**
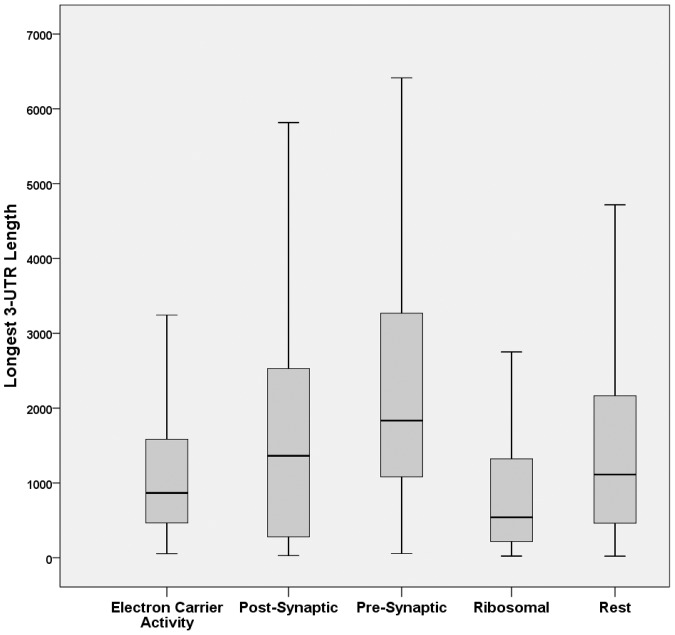
Comparative analysis of longest 3′UTR sequences from synaptic and non-synaptic control transcript groups. Boxplots depicting 3′UTR length of the electron-carrier activity, postsynaptic, presynaptic, ribosomal and rest protein-coding transcripts with the longest 3′UTR sequence.

**Figure 3:**
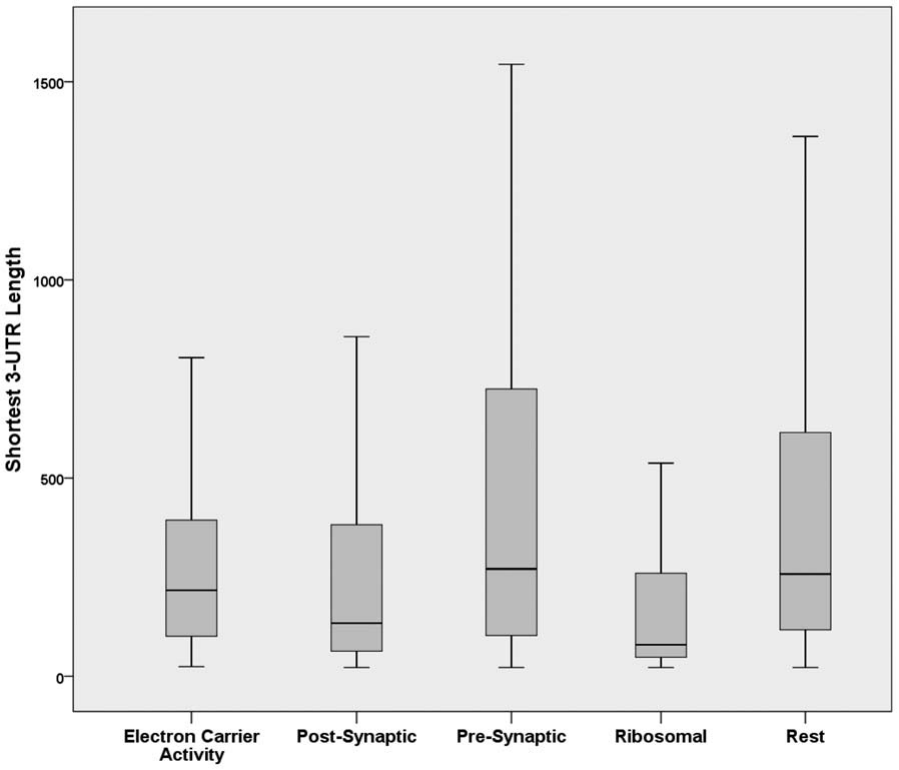
Comparative analysis of shortest 3′UTR sequences from synaptic and non-synaptic control transcript groups. Boxplots depicting 3′UTR length of the electron-carrier activity, postsynaptic, presynaptic, ribosomal and rest protein-coding transcripts with the shortest 3′UTR sequence.

Next, because miRNAs, preferentially, target the 3′UTR of mRNAs [39], [40], it was determined if there was an evolutionary pressure for synaptic mRNAs to maintain long 3′UTRs. For this, the pre- and post- synaptic 3′UTRs were compared with all non-synaptic protein-coding mRNAs. In addition, two randomly selected non-synaptic GO groups with very specialized function in cells, like synapses, were included as controls. These were the ‘structural constituents of ribosome’ (GO:0003735, 152 proteins) and the ‘electron-carrier activity’ (GO:0009055, 158 proteins) groups. Furthermore, given that 3′UTR length may alternate during cellular processes –albeit, predominantly during cell division [41]- both the longest and shortest 3′UTR sequences were investigated, separately. First, the analysis of the longest 3′UTR transcripts revealed that presynaptic transcripts had significantly longer 3′UTRs compared to transcripts from all other categories (p<0.05 in all comparisons, Figure 2, Table 1). On the other hand, postsynaptic transcripts were statistically significantly longer to only ribosomal proteins. Subsequently, the analysis of the shortest 3′UTR transcripts revealed that the presynaptic proteins again possessed significantly longer 3′UTRs than postsynaptic, ribosomal, and electron-carrier activity groups, but not to rest of protein-coding transcripts. In contrast, postsynaptic mRNAs with the shortest 3′UTR isoform had only significantly longer 3′UTRs than ribosomal mRNAs but significantly shorter than electron-carrier activity and rest of protein-coding transcript 3′UTRs (Figure 3, Table 2).

**Table 1:** Comparative analysis of longest 3′UTR sequences between synaptic and non-synaptic control transcript groups.

Longest 3′UTR transcripts	Median 3′UTR length (IQR)	Pair-wise differences (p<0.05)
Presynaptic	1834.5 (2178.8)	*vs:* postsynaptic, Ribosomal, Electron-carrier activity, Rest
Postsynaptic	1363 (2240.5)	*vs:* Ribosomal
Ribosomal	540.5 (1101.75)	*vs:* Presynaptic, Postsynaptic, Electron-carrier activity, Rest
Electron-carrier activity	867 (1117)	*vs:* Presynaptic, Ribosomal
Rest	1112 (1703)	*vs:* Presynaptic, Ribosomal

Median 3′UTR length values (interquartile range) for longest 3′UTR sequences in presynaptic, postsynaptic, ribosomal, electron-carrier activity, and rest protein-coding transcripts. All pairs with statistically significant differences are provided. IQR, interquartile range.

**Table 2:** Comparative analysis of shortest 3′UTR sequences between synaptic and non-synaptic control transcript groups.

Shortest 3′UTR transcripts	Median 3′UTR length (IQR)	Pair-wise differences (p<0.05)
Presynaptic	271(622)	*vs:* Postsynaptic, Ribosomal, Electron-carrier activity
Postsynaptic	134 (319)	*vs:* Ribosomal, Electron-carrier activity, Rest
Ribosomal	79.5 (210.5)	*vs:* Presynaptic, Postsynaptic, Electron-carrier activity, Rest
Electron-carrier activity	217 (293)	*vs:* Presynaptic, Postsynaptic, Ribosomal, Rest
Rest	258 (498)	*vs:* Postsynaptic, Ribosomal, Electron-carrier activity

Median 3′UTR length values (interquartile range) for shortest 3′UTR sequences in presynaptic, postsynaptic, ribosomal, electron-carrier activity, and rest protein coding transcripts. All pairs with statistically significant differences are provided. IQR, interquartile range.

### Prediction of miRNA sites on coding and 3′UTR regions of synaptic mRNAs

Two different algorithms, TargetScan 6.0 and DIANA-microT-CDS updated to the newest mirBase 18 and Ensembl 65 miRNA and mRNA transcript versions, respectively, were used to compile the putative miRNA-mRNA interactions. These algorithms are esteemed to be among the best currently available implementations and can support accurate identification of miRNA binding sites in both 3′UTR and CDS regions. A voting algorithm was implemented, which accepted a predicted interaction only if it was identified by both algorithms. The analysis of pre- and post- synaptic transcripts revealed more than 4,000 and 5,000 miFam-transcript interactions, respectively. Of these, 1,094 interactions, common in both algorithms, were between 211 presynaptic transcripts and 257 miFams while 1,462 interactions were common between 260 postsynaptic transcripts and 296 miFams. Further, all miFam-transcript interactions of both pre- and post- synaptic genes were supported by at least one binding site in the 3′UTR, whereas about a third of predicted interactions involved at least one binding site in the CDS region (Table 3). Subsequent analysis revealed that a set of 38 and 48 miFams could potentially regulate all pre- and post- synaptic transcripts, respectively. Tables S4 and S5 display the results of these analyses.

**Table 3:** Analysis of predicted miFam-transcript interactions.

TS and DmT-CDS predictions	Total interactions	Interactions in 3′UTR	Interactions in CDS
Pre-synaptic	1094	1094	407
Post-synaptic	1462	1462	598

miRNA-transcript interactions predicted from the analysis of 211 pre- and 260 post- synaptic proteins. A voting algorithm was implemented to accept a predicted interaction, only, if both TargetScan v6.0 (TS) and Diana-microT-CDS (DmT-CDS) algorithms identified it.

### Relative position of miRNA binding sites in pre- and post- synaptic 3′UTRs

**Figure 4:**
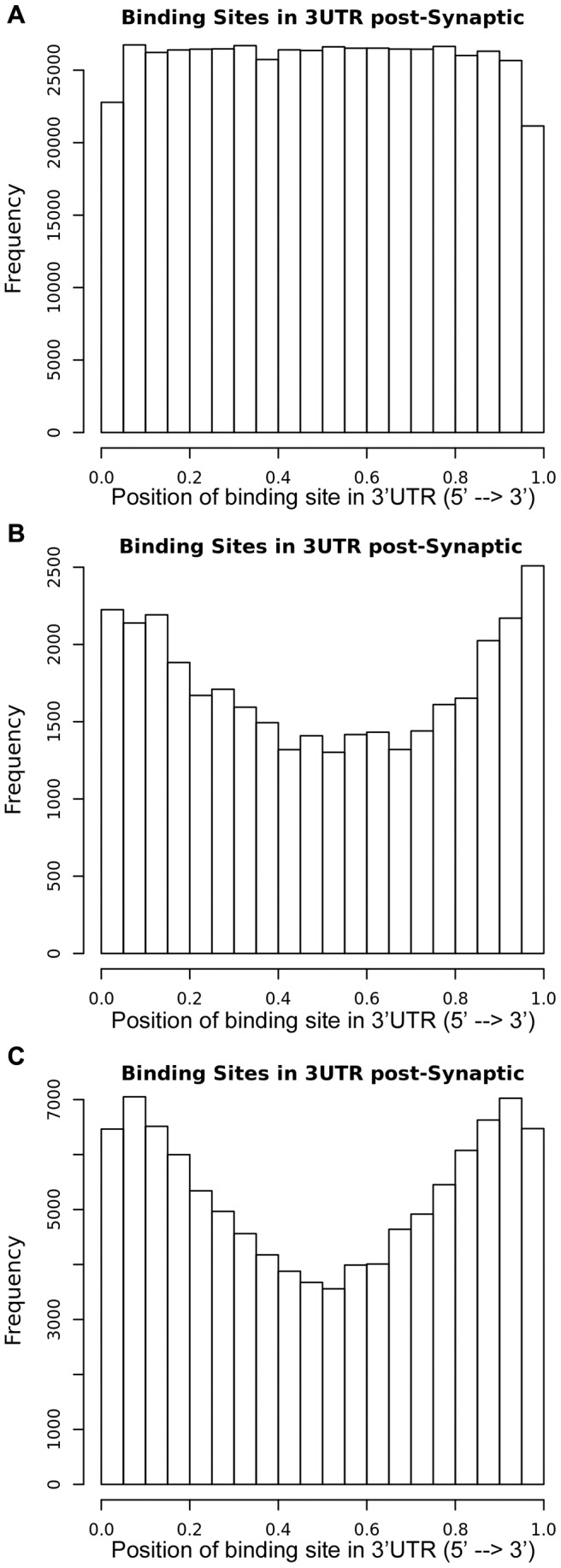
Relative position of miRNA binding sites. Relative position of miRNA binding sites (as percentile of 3′UTR sequence) in postsynaptic genes (**A**) without the use of a threshold in the target prediction results, (**B**) using DIANA-microT-CDS at a threshold of 0.9, and (**C**) using TargetScan v6.0 at a threshold of 0.

The frequency of miRNA binding site positions on the 3′UTR of the pre- and post- synaptic transcripts was, next, estimated. Analysis of both TargetScan 6.0 and DIANA-microT-CDS results revealed that the sites occurred with higher propensity at both the 5′ and 3′ ends of the 3′UTRs sequences. Specifically, when the target prediction results were analyzed without the use of binding quality thresholds, miRNA binding sites were distributed, as expected, uniformly along pre- and post- synaptic 3′UTRs (Figure 4A and data not shown). By filtering DIANA-microT-CDS and TargetScan prediction results with an interaction score threshold of 0.9 and 0, respectively (high precision scores as stated by microT and TargetScan authors), miRNA binding sites in both 5′ and 3′ ends of 3′UTRs appeared to have higher representation than sites in the centre of 3′UTRs (Figures 4B and 4C). The results for the binding sites distributions across the 3′UTRs of presynaptic transcripts were strikingly similar to those of postsynaptic transcripts, with or without the use of binding quality thresholds (data not shown).

### miRNA binding site density in pre- and post- synaptic 3′UTRs

**Figure 5:**
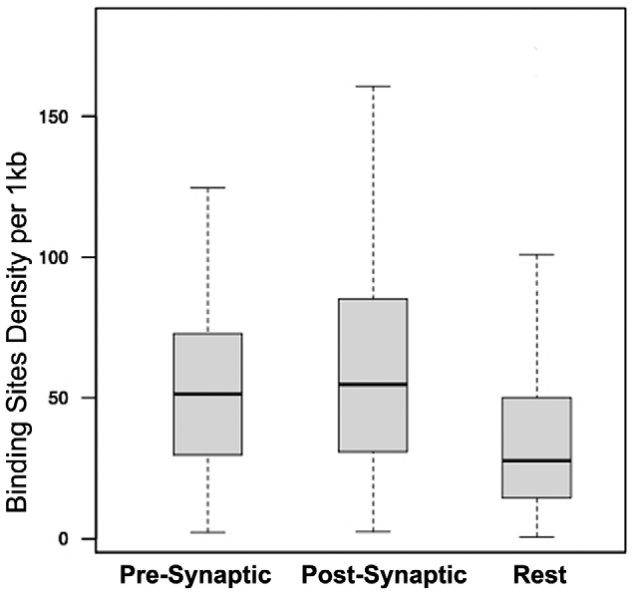
miRNA binding site density in synaptic 3′UTRs. Boxplots presenting binding site densities in the 3′UTR of presynaptic, postsynaptic and the rest of protein-coding transcripts.

Next, the miRNA binding site density in pre- and post- synaptic 3′UTRs was compared to the rest of protein-coding transcripts. The analysis was performed using the longest 3′UTR transcripts. The results revealed that the binding site density in pre- and post- synaptic transcripts was twice higher than the density observed for the rest of protein-coding transcripts. Descriptive as well as inferred statistical results are presented in Figure 5 and Table 4.

**Table 4:** miRNA binding site density in synaptic 3′UTRs.

Categories	Median Binding Site Density (IQR)	Pair-wise differences (p<0.005)
Presynaptic	51.34 (43.07)	*vs:* Rest
Postsynaptic	54.78 (54.1)	*vs:* Rest
Rest	27.77 (35.5)	*vs:* Presynaptic, Postsynaptic

Median values (interquartile range) of the binding site densities in the 3′UTR region of presynaptic, postsynaptic and rest protein-coding transcripts. All pairs with statistically significant differences are provided. IQR, interquartile range.

### Diverse associations between synaptic proteins and miRNAs

Subsequently, analysis of predicted interactions between synaptic proteins and miRNAs was carried out. Thirty-two presynaptic (13%) and forty-three postsynaptic (14%) proteins have had no predicted miRNA binding sites on either CDS or 3′UTR (Table S6). These proteins included cytoskeletal (CFL1, PFN1, PFN3, ACTN3), scaffolding (HOMER3, STX4, SHANK1), vesicular ATPase transporter (ATP6V0A4, ATP6V0C, ATP6V1E2, ATP6V1F), and receptor subunit (CHRNA2, CHRNA5, CHRNA10, CHRNE, GRIK1, GRIK5, GRIN3B) transcripts. The rest 91% of transcripts displayed at least one miRNA binding site on either CDS or 3′UTR.

In principle, one protein can be controlled by more than one miRNA (cooperativity) and one miRNA can target more than one protein (multiplicity) [42]. Cooperativity ensures a more pronounced inhibition and allows multiple miRNA signals to control gene expression. Here, it was found that 47% and 50% of the miRNA-regulated pre- and post- synaptic transcripts were targeted by more than five miRNAs, respectively. Table 5 presents the list of proteins with highest number of predicted miRNA binding sites (for full list, see Tables S7 and S8). They included ANK2 (22 sites), SYNGAP1 (19 sites), SHC18 (20 sites) and SYT4 (17 sites) proteins. Multiplicity is a property arising from relaxed base-pairing between miRNAs and mRNAs. This allows miRNAs to control tenths, if not hundreds, of different transcripts at any given time. Here, 257 and 296 miFams were predicted to target at least one pre- and post- synaptic transcript, respectively. Of these, approximately 11% were found to target more than ten different pre- or post- synaptic transcripts (Tables S9 and S10). Interestingly, the top five miFams with most targets were identical to both pre- and post- synaptic compartments. They were mir-515, mir-506, mir-154, mir-548 and mir-17 (Table 6).

**Table 5:** Synaptic mRNAs with most miRNA interactions.

Protein ID	#	miFam
**Presynaptic**		
SYT4	17	135 550 1294 130 374 379 23 743 15 515 3529 1286 1915 8 876 506 154
CTTNBP2	16	135 497 922 149 15 3119 322 1285 646 103 136 548 449 34 214 154
PTPRD	15	497 204 146 506 19 130 592 17 743 15 25 322 188 148 500
PICALM	14	30 379 1179 17 155 515 181 205 506 548 3158 541 3190 302
SYN2	13	125 3689 30 548 32 449 149 367 25 34 338 506 363
**Postsynaptic**		
ANK2	22	452 290 122 493 647 17 515 197 1205 506 3064 373 103 let-7 449 625 3135 34 1301 214 302 154
SYNGAP1	19	3150 361 939 654 149 197 25 342 608 762 3179 3180 let-7 185 1293 541 491 637 154
SHC4	18	290 374 17 29 515 873 593 4536 506 150 4436 582 182 218 548 3022 302 154
PTPRD	16	497 204 146 506 19 130 592 24 17 743 15 25 322 188 148 500
CADM2	16	575 17 743 515 146 506 125 499 766 548 449 95 664 625 34 154

List of the five pre- and post- synaptic transcripts with most miRNA sites on CDS and 3′UTR as predicted by TargetScan v6.0 and Diana-microT-CDS algorithms. The full list is available in Tables S7 and S8. The miRNAs that target these transcripts are indicated.

**Table 6:** miRNAs with most synaptic mRNA targets.

miFam ID	#	Protein ID
**Presynaptic**		
mir-515	68	SYNCRIP KIF5C LIN7C RTN1 GABRA6 GRM2 KIF5A ACTB TPM3 SEPT2 SEPT7 STX6 DYNLL2 MAP1B VTI1A PPFIA1 ELK1 GRK5 CLCN3 RIMS2 UNC13C STX3 SVOP NPY1R SYNPR ATP6V0A2 STX2 ABL1 SLC17A6 ATP6V1A ZNRF2 STXBP5 MME SCAMP5 EFNB1 SDC2 AP2B1 DCTN1 MYO5A GRM3 CADM1 PFN2 KCTD12 ATP6V1H GABBR2 PPFIBP1 ATP2B1 KIF1B CHL1 STXBP2 SNAP25 ATP6V1C1 ELK3 PTK2 STON1 CASK SYT2 APLP2 RABGAP1 SYT4 CDH2 PICALM PCDH9 SV2B THY1 SLC17A7 SYT1 ATP6V1E1
mir-506	56	SYNCRIP KIF5C GRM1 STXBP6 LIN7C FRAS1 SYN2 SEPT7 LIN7A GAD2 FBXO45 VTI1A GRIK2 GIT2 NPY1R SYNPR STX2 SLC17A6 ATP6V1A GRM8 STXBP5 STX12 RAC1 RIMS1 RAB5C GRM7 SDC2 AP2B1 ERC2 RTN4 CADM1 PFN2 SEPT11 PPFIBP2 PTPRD VAT1 ATP2B1 PXN ELK3 SYPL1 CACNA2D1 ATP6V1G2 SCAMP2 AMPH SYT4 CDH2 NPTN SEPT6 PICALM PCDH9 ATP6V1B2 SYNJ1 KCTD8 NRXN3 VAMP3 SYN1
mir-154	48	SNAP91 LIN7C GRM2 FRAS1 NRXN1 SEPT2 STX6 GAD2 DNM1 SLC30A1 GRK5 GIT2 PLCB4 STXBP3 NPY1R ATP6V0A2 STX2 SLC17A6 ATP6V1A CTTNBP2 GRM8 ZNRF2 STXBP5 SEPT3 MME ACTN4 SYT9 ATP6V1C2 ERC2 GRM3 ACTG1 SLC18A2 KIF1B ELK3 VAMP2 SYPL1 CASK ACTN1 CACNA2D1 NTNG1 CAST SYT4 CDH2 SYNJ1 KCTD8 NRXN3 SYT1 SYN1
mir-548	44	STXBP6 LIN7C RTN1 SYN2 SNAP47 STX17 TPM3 SEPT2 RPH3A DYNLL2 VTI1A GRIK2 GRK5 GIT2 PLCB4 ATP6V0D1 NPY1R SLC17A6 CTTNBP2 SYN3 ZNRF2 STX12 RAC1 RIMS1 RAB5C EFNB1 SDC2 RALA ATP6V1C2 ERC2 GRM3 RTN4 GABBR2 ATP2B1 SYPL1 KIF5B NTNG1 AMPH CDH2 PICALM ATP6V1B2 RAB6C DENND1A VAMP3
mir-17	33	KIF5C STXBP6 KIF5A STX5 GAD2 NCOA2 SLC30A1 GRK5 UNC13C GIT2 CNTNAP1 SVOP SLC17A6 SDC2 RAB5B AP2B1 CADM1 PFN2 SEPT11 GABBR2 PTPRD EFNA1 ERC1 ATP2B1 KIF1B ELK3 RAB5A RABGAP1 SYT10 PICALM SYNJ1 MAP1A SLC17A7
**Postsynaptic**		
mir-515	95	GABRR1 LIN7C SSX2IP TPM3 GRIN2B MARCKS NCAM2 SEPT7 GABRB2 DYNLL2 MAP1B KCND2 OPCML ARHGAP32 GLRB PRKCA EPHB1 SULF1 RYR2 MAP2 GRID2 DLGAP1 MYO5A GRIP1 EPHA5 ADAM10 ANK2 YWHAZ GRIK3 NEO1 GABRA1 ANK3 CHRNA1 CADM3 PTK2 RAP1A CASK RAP1B CLASP1 CAMK2N1 KCNMA1 RAPGEF2 GABRA4 DKK2 GRIA2 CTNND2 PNOC RTN1 GABRA6 EPHB4 ACTB ITPR1 ACTR3 CPEB1 GRB10 SHC4 GRM5 DTNA PPP3R1 CAPRIN2 GRID1 CHRM2 GRIA1 RAPGEF4 PDLIM5 EPHA4 ERBB2IP CRIPT CNTN1 ENAH SDC2 DCTN1 GABRG1 GRIA4 DLG1 GRM3 CADM1 CSNK2A1 TANC1 EPHA7 PFN2 CNKSR2 KCTD12 GABBR2 HSPA12A CHL1 DLG2 CADM2 NR3C1 PPP1R9A CDH2 PCDH9 PICALM GDA NLGN4X
mir-506	76	GRM1 LIN7C SSX2IP PRKCE SEPT7 LIN7A KCND2 GLRB PPP1CC EPHB1 MAP2 DLG3 SPTBN1 PCDH8 DLGAP1 GRM7 RTN4 GRIP1 ADAM10 CHRNA3 ANK2 NEO1 CAMK2D GABRQ SPOCK1 LRRTM3 GRIA3 CAMK2N1 LRRTM4 DKK3 NLGN4Y DKK2 GRIA2 LRRC4C CTNND2 FRAS1 CPEB1 SHC4 GLRA2 PPP3R1 GRID1 LRRC7 GRIK2 PDLIM5 ACTR2 CLSTN2 EPHA4 MAGI2 GKAP1 CNTN1 SDC2 GRIA4 DLG1 CADM1 TANC1 EPHA7 PFN2 CNKSR2 GABRG2 PTPRD HSPA12A SHANK2 DLG2 CADM2 NR3C1 PPP1R9A CDH2 PCDH9 PICALM CNTN5 GABRB3 KCTD8 STRN GDA CHRM1 NLGN4X
mir-548	76	CLSTN3 COL3A1 LIN7C MTMR2 TPM3 CDK5R1 RPH3A DYNLL2 CAV2 GPHN NLGN3 KCND2 GABRB1 GLRB PPP1CC EPHB1 SULF1 VCL MAP2 PLCB4 GRID2 NCAM1 RTN4 GRIP1 ADAM10 YWHAZ NEO1 GABRA3 GABRA1 CTNNB1 RAP1B LRRTM3 GRIA3 KCNMA1 LRRTM4 DKK3 NLGN4Y HOMER1 CAMK2B AXIN2 DKK2 DSCAM LRRC4C CTTN ADD1 GABRP RTN1 EPHB4 GRB10 SHC4 GLRA2 PPP3R1 LRRC7 GRIA1 RAPGEF4 GRIK2 ACTR2 EPHA4 MAGI2 BAIAP2 CRIPT CNTN1 NTM SDC2 DLG1 GRM3 CNKSR2 LGI1 GABBR2 CTNNA1 CADM2 LRRC4B GRIPAP1 CDH2 PICALM CNTN5
mir-154	69	LIN7C SSX2IP MARCKS ARF3 CAV2 DNM1 KCND2 GLRB EPHB1 SULF1 VCL MAP2 PLCB4 GRID2 CBLN4 DLG3 GABRA5 PCDH8 DLGAP1 CHRNA7 GOPC ADAM10 ANK2 GABRA1 SHANK3 ANK3 CTNNB1 CASK RAP1B LRRTM3 GRIA3 CAMK2N1 KCNMA1 DKK3 LRFN5 AXIN2 DKK2 GRIA2 LRRC4C SYNGAP1 SNAP91 FRAS1 CPEB1 SHC4 GLRA2 PPP3R1 WIF1 SYNE1 LRRC7 GRIA1 SLITRK1 EPHA4 MAGI2 ACTN4 NTM DLG1 GRM3 CSNK2A1 TANC1 ACTG1 GABRG2 CADM2 NR3C1 ACTN1 CDH2 CNTN5 GABRB3 KCTD8 NLGN4X
mir-17	43	SSX2IP GABRB2 KCND2 NCOA2 GLRB CNTNAP1 GRID2 DLGAP1 CHRNA7 EPHA5 GRIN1 ANK2 NEO1 CAMK2D GLRA3 CAMK2N1 KCNMA1 MAP1A GABRA4 SHC4 NYX PPP3R1 CAPRIN2 CHRM2 RAPGEF4 SLITRK1 EPHA4 GABRE CRIPT ENAH SDC2 GABRG1 GRIA4 CADM1 TANC1 EPHA7 PFN2 CNKSR2 GABBR2 PTPRD DLG2 CADM2 PICALM

List of the five miRNA families with most pre- and post- synaptic targets predicted by both TargetScan v6.0 and Diana-microT-CDS algorithms. The full list is available in Tables S9 and S10. MiFam members' classification is shown in Table S11. The targeted transcripts are indicated.

**Figure 6:**
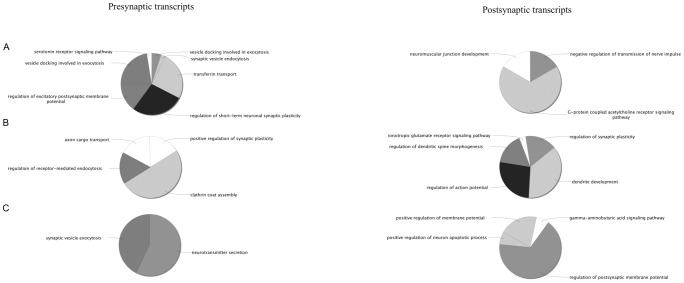
Gene ontology-enriched categories of synaptic mRNAs with least and most miRNA predicted interactions. GO-enriched categories determined by ClueGo in (**A**) transcripts with zero or one predicted miRNA site (**B**) transcripts with over eight different miRNA sites and (**C**) common to both transcript groups with least and most miRNA sites. A two-sided hypergeometric test yielded the enrichment for GO terms. Benjamini-Hochberg correction for multiple testing controlled the *P*-values.

Moreover, the GO biological processes of synaptic proteins with no or only one predicted miRNA binding site were compared to those with over eight (to compare approximately equal number of proteins) predicted miRNA sites. Using ClueGO analysis, presynaptic transcripts with no or only one miRNA binding site were enriched for ‘energy coupled proton transport, against electrochemical gradient’ and ‘vesicle docking involved in exocytosis’ GO terms while those with more than eight miRNA binding sites were enriched for ‘clathrin coat assembly’ and ‘axon cargo transport’ GO terms. Common biological process categories were ‘synaptic vesicle exocytosis’ and ‘neurotransmitter secretion’ (Figure 6). With respect to postsynaptic transcripts, those with no or only one miRNA binding site, were enriched for ‘G-protein coupled acetylcholine receptor signaling’ GO term while those with eight or more sites were enriched for ‘dendrite development’ and ‘action potential regulation’ GO terms. Common biological category included ‘the regulation of postsynaptic membrane potential’ (Figure 6).

### Construction of interaction map between miRNAs and corresponding targets

Because there is high degree of cooperativity and multiplicity at synapses, it was determined if there exists a set of miRNAs that could potentially regulate the majority of the synaptic proteins. This information is relevant because deregulation of the particular miRNAs would, likely, result in alteration of synaptic function and, thus, may prove ideal therapeutic targets for the treatment of psychiatric diseases.

**Figure 7:**
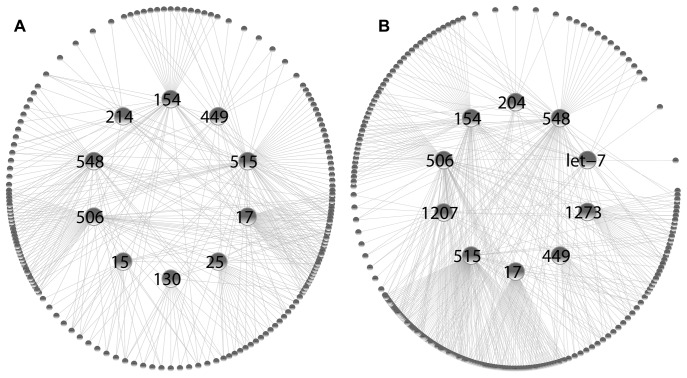
Interaction map between miRNAs and predicted pre- and post- synaptic mRNA targets. A set of ten miRNAs regulated 77% and 80% of pre- and post- synaptic transcripts, respectively. A script was used to identify the combination of miRNAs that were found to interact with the maximum number of synaptic mRNAs. Direct interactions between miRNAs and mRNAs are depicted with grey lines.

Hence, a script was implemented to identify the minimum cover set of miFams regulating most pre- and post- synaptic transcripts. Two groups of ten miRNA families were, subsequently, found to regulate 77.2% and 79.6% of pre- and post- synaptic transcripts, respectively (Figure 7). The analysis, further, revealed a varied degree of cooperativity and selectivity between these miFams. Mir-17 family was found to have almost no connection to the target niches of other miFams in both pre- and post- synaptic compartments; this was also the case for mir-515 and mir-1273 families in postsynaptic terminals. In contrast, mir-548 and mir-154 had radiating connections to most other miFams. Among the miFams, mir-17, -515, -154 and -506 had most mRNA targets in both pre- and post- synaptic compartments, partly reflecting the large number of miRNA members.

### Pathway Analysis

Following the prediction of the relevant miFams for synaptic mRNA regulation, their overall biological function was determined. For this, DIANA-mirPath v2.0 was used to annotate all predicted and experimentally validated targets of the selected miFams in molecular KEGG pathways. Table 7 depicts the enriched KEGG pathways for mir-154. Several neuronal categories were identified that included ‘prion diseases’ (p<1×10^−16^), ‘neurotrophin signaling pathway’ (p<2.8×10^−5^), ‘axon guidance’ (p<6.2×10^−5^) and ‘long-term potentiation’ (p<0.007) confirming mir-154's association with neuronal function. The transcripts targeted by mir-154 family in each of the aforementioned pathways are shown in Figures S3, S4, S5, S6, S7. Other relevant enriched categories included ‘TGF-β (p<1×10^−16^) and Wnt (p<9.6×10^−8^) signaling pathways’, ‘glycosaminoglycan biosynthesis’ (p<1×10^−16^), ‘focal adhesion’ (p<7×10^−7^) and ‘endocytosis’ (p<4.7×10^−6^). Similar results were obtained from the analysis of the other top synaptic miFams for which the data can be found in Tables S12, S13, S14, S15, S16, S17, S18, S19, S20, S21, S22, S23, S4, and S25.

**Table 7:** Enriched KEGG pathways for predicted mir-154 family targets.

KEGG pathway	*P-*value	# genes	# miRNAs
Glycosaminoglycan biosynthesis – heparin sulfate	<1×10^−16^	9	9
TGF-beta signaling pathway	<1×10^−16^	35	21
Prion diseases	5.4×10^−14^	7	14
ECM-receptor interaction	9.0×10^−12^	22	20
Glycosaminoglycan biosynthesis - chondroitin sulfate	6.6×10^−11^	5	6
N-Glycan biosynthesis	1.8×10^−09^	15	19
Wnt signaling pathway	9.6×10^−08^	53	23
Mucin type O-Glycan biosynthesis	1.1×10^−07^	6	10
Lysine degradation	1.1×10^−07^	16	16
Focal adhesion	7.0×10^−07^	69	24
Adherens junction	7.9×10^−07^	33	18
Pathways in cancer	4.0×10^−06^	99	22
Endocytosis	4.7×10^−06^	58	23
Neurotrophin signaling pathway	2.8×10^−05^	43	23
Adipocytokine signaling pathway	3.0×10^−05^	25	19
Axon guidance	6.2×10^−05^	41	20
Metabolism of xenobiotics by cytochrome P450	0.0004	8	9
Biosynthesis of unsaturated fatty acids	0.0004	6	6
Cytokine-cytokine receptor interaction	0.0006	52	18
ErbB signaling pathway	0.0007	28	20
Renal cell carcinoma	0.0014	26	18
Gap junction	0.0048	26	17
Vascular smooth muscle contraction	0.0067	31	20
Long-term potentiation	0.0071	26	20
Drug metabolism - cytochrome P450	0.014	10	11
Glioma	0.016	22	20
Leukocyte transendothelial migration	0.016	32	20
Maturity onset diabetes of the young	0.023	8	8
Cell adhesion molecules (CAMs)	0.030	36	19
Regulation of actin cytoskeleton	0.030	60	22

DIANA-miRPath v2.0 was used to predict the KEGG pathways of mir-154 family targets. The target prediction threshold was set at 0.85. Benjamini-Hochberg [38] correction for multiple testing controlled the *P*-values.

## Discussion

miRNAs have been recognized as essential for neuron development and differentiation on the basis of original research in which the miRNA processing enzymes Dicer or Dgcr8 has been knocked-down [43], [44], [45]; however, the role they have in homeostasis of mature neurons and, in particular, synaptic function remains poorly understood. The current challenge is to elucidate the biological functions of individual miRNAs in neurons and discover the interaction networks they control. With respect to the synapse, it is still unclear which miRNAs are critical for its function and are not by-standers, the extent into which they control the different synaptic processes and the regulatory networks they participate.

Towards this, a reverse bioinformatics approach has been undertaken to identify miRNA roles at the synapse. An excess of 500 transcripts, representing the different synaptic molecular categories at pre- and post- synaptic terminals were analyzed for their 3′ UTR length, miRNA binding sites distribution and density. In the end, the miRNAs with most synaptic targets were examined for overall function.

At first, length analysis of longest 3′UTR transcripts revealed that presynaptic proteins had, significantly, longer 3′UTRs compared to all other transcripts including postsynaptic. The tendency to have relative longer 3′UTRs remained when analysis of the shortest 3′UTR isoforms of presynaptic proteins was carried out. In contrast, postsynaptic transcripts revealed a significant drop in 3′UTR length between longest and shortest 3′UTR isoforms. These results indicated that presynaptic proteins maintained a relative long 3′UTR for enhanced miRNA regulation irrespective of 3′UTR length fluctuations while postsynaptic proteins possessed a broader spectrum of 3′UTR lengths to avert miRNA regulation under specific conditions. Currently, we know little of what determines 3′UTR length variation in neurons but one report indicated that both short and long forms coexist with the longer form determining localization in dendrites [46]. Another report indicated that longer 3′UTR forms appear with aging as a result of weakened mRNA polyadenylation activity [47].

Subsequently, the miRNA sites of each synaptic protein were determined. Analyzing the data, it was found that miRNA sites in synaptic 3′UTRs were distributed with higher propensity at both the 5′ and 3′ ends of 3′UTRs. These results, likely, reflected better silencing efficiency at these ends as target sites in the middle of 3′UTR have been found to be less efficient for RNAi regulation [48]. Similar distributions have been, previously, reported from analysis of total mRNAome [49]. Next, the miRNA binding site density in synaptic 3′UTRs was compared to rest of protein-coding transcripts. It was found that both pre- and post- synaptic transcripts had similar site densities that were twice as dense as the rest of protein-coding transcripts. These data confirmed the higher propensity of synaptic transcripts, irrespectively of their length, to be miRNA targets.

This study, also, revealed that more than 90% of synaptic transcripts were predicted to have at least one miRNA binding site with, approximately, half being targeted by more than five miRNAs. These findings indicated that miRNA regulation is widespread among synaptic proteins and that multiple miRNAs ensure tight control of synaptic mRNA expression. Further, the miRNA families with most targets - mir-515, mir-506, mir-154, mir-548 and mir-17 - were identical to both pre- and post- synaptic compartments indicating coordinated miRNA regulation of mRNA expression at the synapse. This is maybe expected since for most neurons activity levels at their dendritic and axon termini need to be coordinated. By comparing the synaptic proteins according to the number of miRNA binding sites they possessed, it was revealed that different synaptic processes are prone differently to miRNA regulation. For instance, proteins involved in synaptic vesicle maturation were least associated with miRNA control while those involved with dendritic development and the regulation of action potentials had eight or more predicted miRNA binding sites.

Finally, an interaction map was used to unveil the intricate associations between the ten most relevant miFams and synaptic transcripts. Accommodating the 80% of pre- and post- synaptic transcripts it revealed that a) six miRNA families were common to both synaptic compartments: mir-154, mir-449, mir-515, mir-17, mir-506, mir-548, while four were different: mir-25, mir-130, mir-15, mir-214 for pre- and mir-204, mir-1273, mir-1207, let-7 for post- synaptic termini. Intriguingly, some of these miFams, like mir-17 had distinct target niches, implicating control over a discrete functional group, while others, like mir-154, had radiating projections to most other miFam niches, possibly reflecting hierarchical or coordinated control of synaptic protein expression between groups.

From the lists of most relevant miFams nearly half have, still, only been identified in or are specific for primates. These are miFams mir-515, mir-548, mir-1273, and mir-1207. With respect to mir-506 family only two out of nine miRNA members have so far been identified in mouse; these are mir-511 and mir-509. On the other hand, mir-154, mir-449, mir-25, mir-214, mir-17, mir-449 and let-7 families have all got corresponding miRNAs in mouse. If these findings are confirmed, and no analogous miRNAs are found in lower mammals, it is reasonable to speculate that differences in cognition between high-level primates and non-primate mammals could, in part, be attributed to the presence or not of these primate-specific miRNAs.

Current knowledge on the function of the specified miRNAs is limited, nevertheless informative. Most, originates from research on deregulated miRNAs in patients with neurological disorders. Hence, research in schizophrenia, which is a debilitating psychotic disorder affecting neural circuitry and synaptic function, has found that most members of miFams identified here, as deregulated (reviewed in [50]). These included, mir-154, mir-381, mir-382, mir-323, mir-409, mir-487, mir-449, mir-548, mir-519, mir-517, mir-520, mir-518, mir-25, mir-92, mir-17, mir-93, mir-106, mir-20, mir-512, mir-509, mir-510, mir-548 and let-7e,d,f. Further, some of these miRNA like mir-17, mir-517 and mir-20 have, also, been found to be deregulated in depressed suicide subjects [51] while others like mir-449, mir-92, mir-323, mir-410, mir-17, mir-154, mir-409, mir-25 and let-7 have been differentially expressed during aging of murine brains [52]. Moreover, two of these miRNAs, mir-92 and mir-25, have been shown to be deregulated during song-specific habituation in the auditory forebrain [53].

To better understand the biological function of the identified miFams, DIANA-mirPath v2.0 was employed to reveal the enriched KEGG pathway categories of all – not just synaptic- their predicted target mRNA transcripts. As would be expected for miRNAs with dynamic roles in neurons, several enriched neuronal categories characterized most of these miFams. They included ‘neurotrophin signaling’, ‘axon guidance’, ‘long-term potentiation or long-term depression’, ‘circadian rhythms’, and ‘SNARE interactions in vesicular transport’. These results provided alternative evidence that the top miFams identified in this study are relevant to synaptic function and shed further light into their overall biological function.

Currently, we know of several miRNAs that have important roles at synapses. One such miRNA is mir-34 that has been found to regulate neurite outgrowth, spinal morphology, and synaptic targets [54], [55]; moreover, it was shown to be down-regulated in Alzheimer's disease [56], upregulated in schizophrenia, bipolar disorders [57], and seizure-induced death [58] and was put forward as a target to treat dementias [59] and stress-induced anxiety [60]. Mir-34 appeared in the top fifteen miFams of this study with 12 and 15 pre- and postsynaptic targets, respectively. Another miRNA with important synaptic roles is mir-132, a CREB-induced microRNA that promotes dendrite length, arborization, and spine density in hippocampal and olfactory bulb neurons [61] [62]. It was, also, found to regulate ocular dominance plasticity [63] and circadian clock period and entrainment [64]. Mir-132 was predicted to control 9 synaptic targets in current study. Mir-188, another synaptic active miRNA, was recently shown to be upregulated during long-term potentiation and rescue the reduction in dendritic spine density induced by Nrp-2 expression [65]. It was, also, found to be deregulated after sciatic nerve transection [66]. Mir-188 appeared in the top twenty miFams of this study with 10 pre- and 13 postsynaptic targets, respectively. MiR-134 and mir-138 have seemingly opposite function to mir-132 and mir-188 since they were shown to, negatively, regulate the size of dendritic spines via inhibition of Limk1 translation and acyl protein thioesterase 1 (APT1), respectively [67], [68]. Further, mir-134 action rendered animals refractory to seizures and hippocampal injury caused by status epilepticus [69]. With respect to current study, mir-134 was found not to have synaptic targets while mir-138 was predicted to have only 1 and 2 targets in pre- and post- synaptic terminals, respectively. Collectively, based on this small sample of experimentally verified synaptic miRNAs, it can be inferred that the number of predicted synaptic targets may not necessarily reflect the importance of a particular miRNA at the synapse.

In summary, we used computational approaches to identify and, in part, characterize the miRNA regulatory landscape at the synapse. Based on the expression levels of these miRNAs as a function of brain area, activity or age, we expect the strength of regulation applied at each synaptic protein to vary between neuronal populations at any one time. Further characterization of these miRNAs should improve our understanding of synaptic activity and neuronal function, shed light into the cognitive differences between primates and non-primate mammals and uncover novel therapeutic targets for psychiatric diseases.

## Supporting Information

Figure S1 **ClueGo GO functional analysis of presynaptic proteins.** (**A**) Functionally grouped network with GO terms as nodes linked based on their kappa score level (>0.3), were only the label of the most significant term per group is shown. Functionally related GO terms are adjacent to each other. Not grouped GO terms are shown in white. (**B**) Overview chart with enriched functional GO groups. A two-sided hypergeometric test yielded the enrichment for GO terms. Benjamini-Hochberg correction for multiple testing controlled the *P*-values.
(XMLNS)

Figure S2 **ClueGo GO functional analysis of postsynaptic proteins.** (**A**) Functionally grouped network with GO terms as nodes linked based on their kappa score level (>0.3), were only the label of the most significant term per group is shown. Functionally related GO terms are adjacent to each other. Not grouped GO terms are shown in white. (**B**) Overview chart with enriched functional GO groups. A two-sided hypergeometric test yielded the enrichment for GO terms. Benjamini-Hochberg correction for multiple testing controlled the *P*-values.
(XMLNS)

Figure S3 **mir-154 family targets in the long term potentiation KEGG pathway.** DIANA-miRPath v2.0 was used to visualize mir-154 predicted targets in the enriched long term potentiation KEGG pathway. The target prediction threshold was set at 0.85. Benjamini-Hochberg [38] correction for multiple testing controlled the *P*-values.
(XMLNS)

Figure S4 **mir-154 family targets in the axon guidance KEGG pathway.** DIANA-miRPath v2.0 was used to visualize mir-154 predicted targets in the enriched axon guidance KEGG pathway. The target prediction threshold was set at 0.85. Benjamini-Hochberg [38] correction for multiple testing controlled the *P*-values.
(XMLNS)

Figure S5 **mir-154 family targets in the neurotrophin KEGG pathway.** DIANA-miRPath v2.0 was used to visualize mir-154 predicted targets in the enriched neurotrophin KEGG pathway. The target prediction threshold was set at 0.85. Benjamini-Hochberg [38] correction for multiple testing controlled the *P*-values.
(XMLNS)

Figure S6 **mir-154 family targets in the focal adhesion KEGG pathway.** DIANA-miRPath v2.0 was used to visualize mir-154 predicted targets in the enriched focal adhesion KEGG pathway. The target prediction threshold was set at 0.85. Benjamini-Hochberg [38] correction for multiple testing controlled the *P*-values.
(XMLNS)

Figure S7 **mir-154 family targets in the regulation of actin cytoskeleton KEGG pathway.** DIANA-miRPath v2.0 was used to visualize mir-154 predicted targets in the enriched actin cytoskeleton KEGG pathway. The target prediction threshold was set at 0.85. Benjamini-Hochberg [38] correction for multiple testing controlled the *P*-values.
(XMLNS)

Table S1 List of synaptic proteins analyzed. 242 and 304 pre- and post- synaptic proteins a) amassed from an extensive literature review and b) curated from GO synapse subcategories were analyzed for miRNA regulation, respectively.
(XLSX)

Table S2 Analysis of presynaptic proteins. ClueGo analysis of GO terms enriched among presynaptic proteins. A two-sided hypergeometric test yielded the enrichment for GO terms. Benjamini-Hochberg correction for multiple testing controlled the *P*-values.
(XLSX)

Table S3 Analysis of postsynaptic proteins. ClueGo analysis of GO terms enriched among postsynaptic proteins. A two-sided hypergeometric test yielded the enrichment for GO terms. Benjamini-Hochberg correction for multiple testing controlled the *P*-values.
(XLSX)

Table S4 List of predicted miRNA-presynaptic transcript interactions. miRNA predictions in both CDS and 3′UTR of each presynaptic protein using TargetScan v6.0 or Diana-microT-CDS algorithms.
(XLSX)

Table S5 List of predicted miRNA-postsynaptic transcript interactions. miRNA predictions in both CDS and 3′UTR of each postsynaptic protein using TargetScan v6.0 or Diana-microT-CDS algorithms.
(XLSX)

Table S6 List of synaptic proteins with no predicted miRNA targets. List of pre- and post- synaptic transcripts with no shared miRNA predictions between TargetScan v6.0 and Diana-microT-CDS algorithms.
(XLSX)

Table S7 miRNA predictions for each presynaptic protein. Full list of presynaptic transcripts targeted by multiple miRNAs as predicted by both TargetScan v6.0 and Diana-microT-CDS algorithms. Transcripts with most miRNA binding sites are shown in ascending order.
(XLSX)

Table S8 miRNA predictions for each postsynaptic protein. Full list of postsynaptic transcripts targeted by multiple miRNAs as predicted by both TargetScan v6.0 and Diana-microT-CDS algorithms. Transcripts with most miRNA binding sites are shown in ascending order.
(XLSX)

Table S9 List of miRNA presynaptic targets. Full list of miRNA presynaptic targets as predicted by both TargetScan v6.0 and Diana-microT-CDS algorithms. miRNAs with highest number of synaptic targets is shown in an descending order.
(XLSX)

Table S10 List of miRNA postsynaptic targets. Full list of miRNA postsynaptic targets as predicted by both TargetScan v6.0 and Diana-microT-CDS algorithms. miRNAs with highest number of synaptic targets is shown in a descending order.
(XLSX)

Table S11 miFam members' classification. List of all human miRNAs classified into families according to miRBase 18.
(XLSX)

Table S12 Enriched KEGG pathways of mir-154 family targets. DIANA-miRPath v2.0 was used to predict all enriched KEGG pathways of mir-154 family targets. The target prediction threshold was set at 0.85. Benjamini-Hochberg [38] correction for multiple testing controlled the *P*-values.
(XLSX)

Table S13 Enriched KEGG pathways of mir-204 family targets. DIANA-miRPath v2.0 was used to predict all enriched KEGG pathways of mir-204 family targets. The target prediction threshold was set at 0.85. Benjamini-Hochberg [38] correction for multiple testing controlled the *P*-values.
(XLSX)

Table S14 Enriched KEGG pathways of let-7 family targets. DIANA-miRPath v2.0 was used to predict all enriched KEGG pathways of let-7 family targets. The target prediction threshold was set at 0.85. Benjamini-Hochberg [38] correction for multiple testing controlled the *P*-values.
(XLSX)

Table S15 Enriched KEGG pathways of mir-515 family targets. DIANA-miRPath v2.0 was used to predict all enriched KEGG pathways of mir-515 family targets. The target prediction threshold was set at 0.85. Benjamini-Hochberg [38] correction for multiple testing controlled the *P*-values.
(XLSX)

Table S16 Enriched KEGG pathways of mir-25 family targets. DIANA-miRPath v2.0 was used to predict all enriched KEGG pathways of mir-25 family targets. The target prediction threshold was set at 0.85. Benjamini-Hochberg [38] correction for multiple testing controlled the *P*-values.
(XLSX)

Table S17 Enriched KEGG pathways of mir-130 family targets. DIANA-miRPath v2.0 was used to predict all enriched KEGG pathways of mir-130 family targets. The target prediction threshold was set at 0.85. Benjamini-Hochberg [38] correction for multiple testing controlled the *P*-values.
(XLSX)

Table S18 Enriched KEGG pathways of mir-17 family targets. DIANA-miRPath v2.0 was used to predict all enriched KEGG pathways of mir-17 family targets. The target prediction threshold was set at 0.85. Benjamini-Hochberg [38] correction for multiple testing controlled the *P*-values.
(XLSX)

Table S19 Enriched KEGG pathways of mir-15 family targets. DIANA-miRPath v2.0 was used to predict all enriched KEGG pathways of mir-15 family targets. The target prediction threshold was set at 0.85. Benjamini-Hochberg [38] correction for multiple testing controlled the *P*-values.
(XLSX)

Table S20 Enriched KEGG pathways of mir-584 family targets. DIANA-miRPath v2.0 was used to predict all enriched KEGG pathways of mir-584 family targets. The target prediction threshold was set at 0.85. Benjamini-Hochberg [38] correction for multiple testing controlled the *P*-values.
(XLSX)

Table S21 Enriched KEGG pathways of mir-1207 family targets. DIANA-miRPath v2.0 was used to predict all enriched KEGG pathways of mir-1207 family targets. The target prediction threshold was set at 0.85. Benjamini-Hochberg [38] correction for multiple testing controlled the *P*-values.
(XLSX)

Table S22 Enriched KEGG pathways of mir-506 family targets. DIANA-miRPath v2.0 was used to predict all enriched KEGG pathways of mir-506 family targets. The target prediction threshold was set at 0.85. Benjamini-Hochberg [38] correction for multiple testing controlled the *P*-values.
(XLSX)

Table S23 Enriched KEGG pathways of mir-214 family targets. DIANA-miRPath v2.0 was used to predict all enriched KEGG pathways of mir-214 family targets. The target prediction threshold was set at 0.85. Benjamini-Hochberg [38] correction for multiple testing controlled the *P*-values.
(XLSX)

Table S24 Enriched KEGG pathways of mir-1273 family targets. DIANA-miRPath v2.0 was used to predict all enriched KEGG pathways of mir-1273 family targets. The target prediction threshold was set at 0.85. Benjamini-Hochberg [38] correction for multiple testing controlled the *P*-values.
(XLSX)

Table S25 Enriched KEGG pathways of mir-449 family targets. DIANA-miRPath v2.0 was used to predict all enriched KEGG pathways of mir-449 family targets. The target prediction threshold was set at 0.85. Benjamini-Hochberg [38] correction for multiple testing controlled the *P*-values.
(XLSX)
