# Supplementary figures and images for: miRNA Regulons Associated with Synaptic Function
Source: PLoS One. 2012 Oct 8;7(10):e46189. doi: 10.1371/journal.pone.0046189 (PMC3468272; doi:10.1371/journal.pone.0046189)

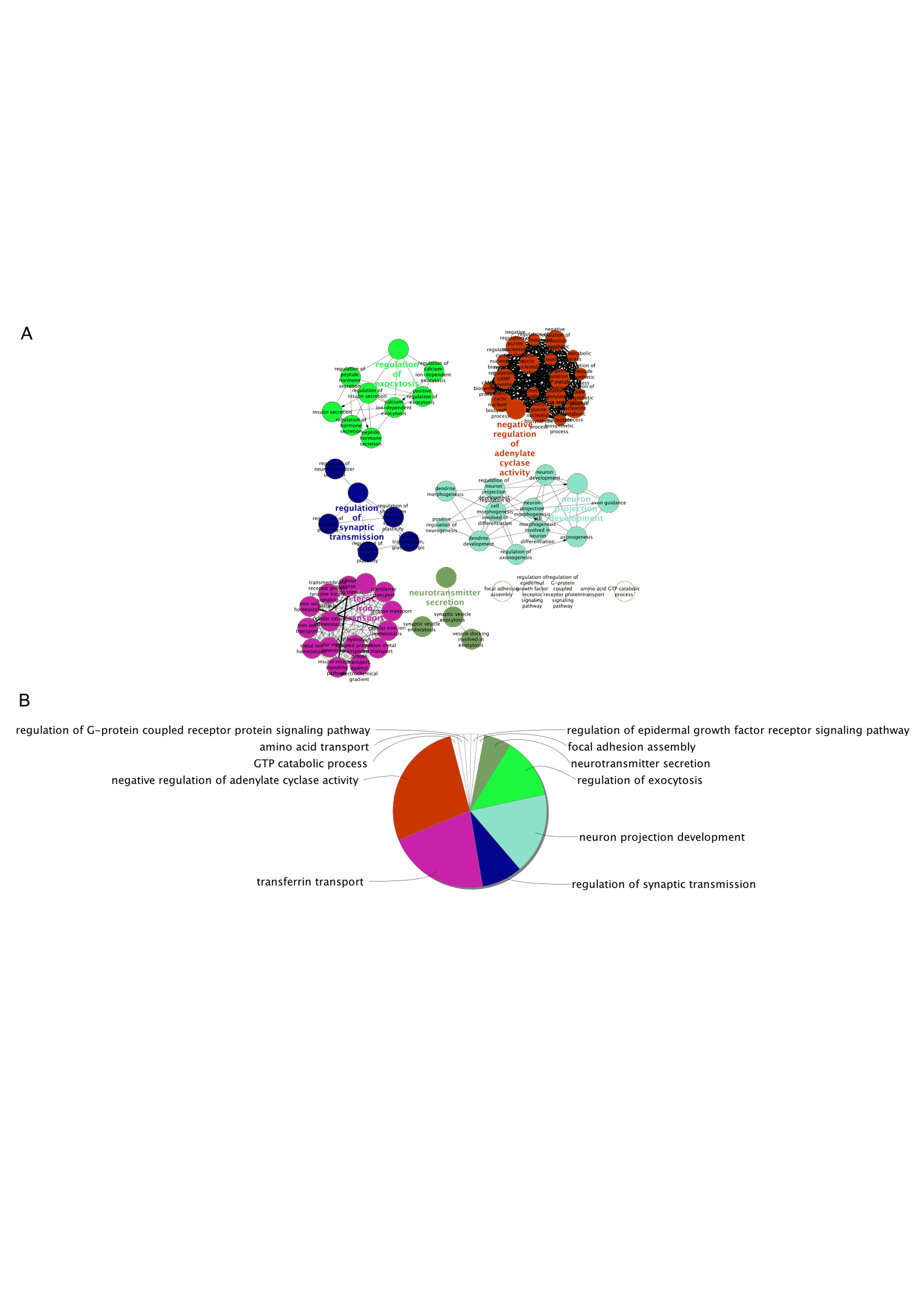

Supplement: Figure S1 — ClueGo GO functional analysis of presynaptic proteins. (A) Functionally grouped network with GO terms as nodes linked based on their kappa score level (>0.3), were only the label of the most significant term per group is shown. Functionally related GO terms are adjacent to each other. Not grouped GO terms are shown in white. (B) Overview chart with enriched functional GO groups. A two-sided hypergeometric test yielded the enrichment for GO terms. Benjamini-Hochberg correction for multiple testing controlled the P-values. (XMLNS) [file pone.0046189.s001.xml]

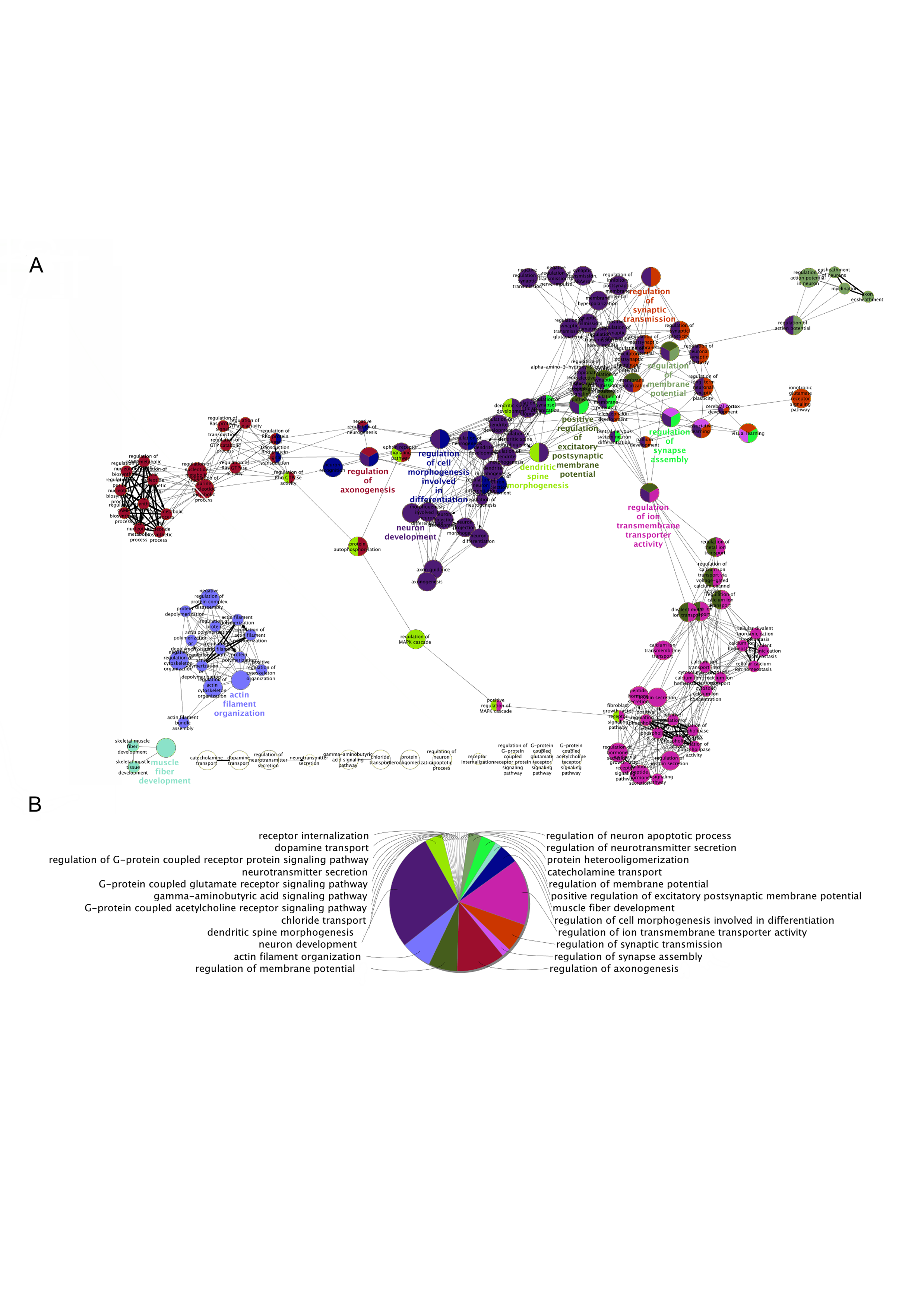

Supplement: Figure S2 — ClueGo GO functional analysis of postsynaptic proteins. (A) Functionally grouped network with GO terms as nodes linked based on their kappa score level (>0.3), were only the label of the most significant term per group is shown. Functionally related GO terms are adjacent to each other. Not grouped GO terms are shown in white. (B) Overview chart with enriched functional GO groups. A two-sided hypergeometric test yielded the enrichment for GO terms. Benjamini-Hochberg correction for multiple testing controlled the P-values. (XMLNS) [file pone.0046189.s002.xml]

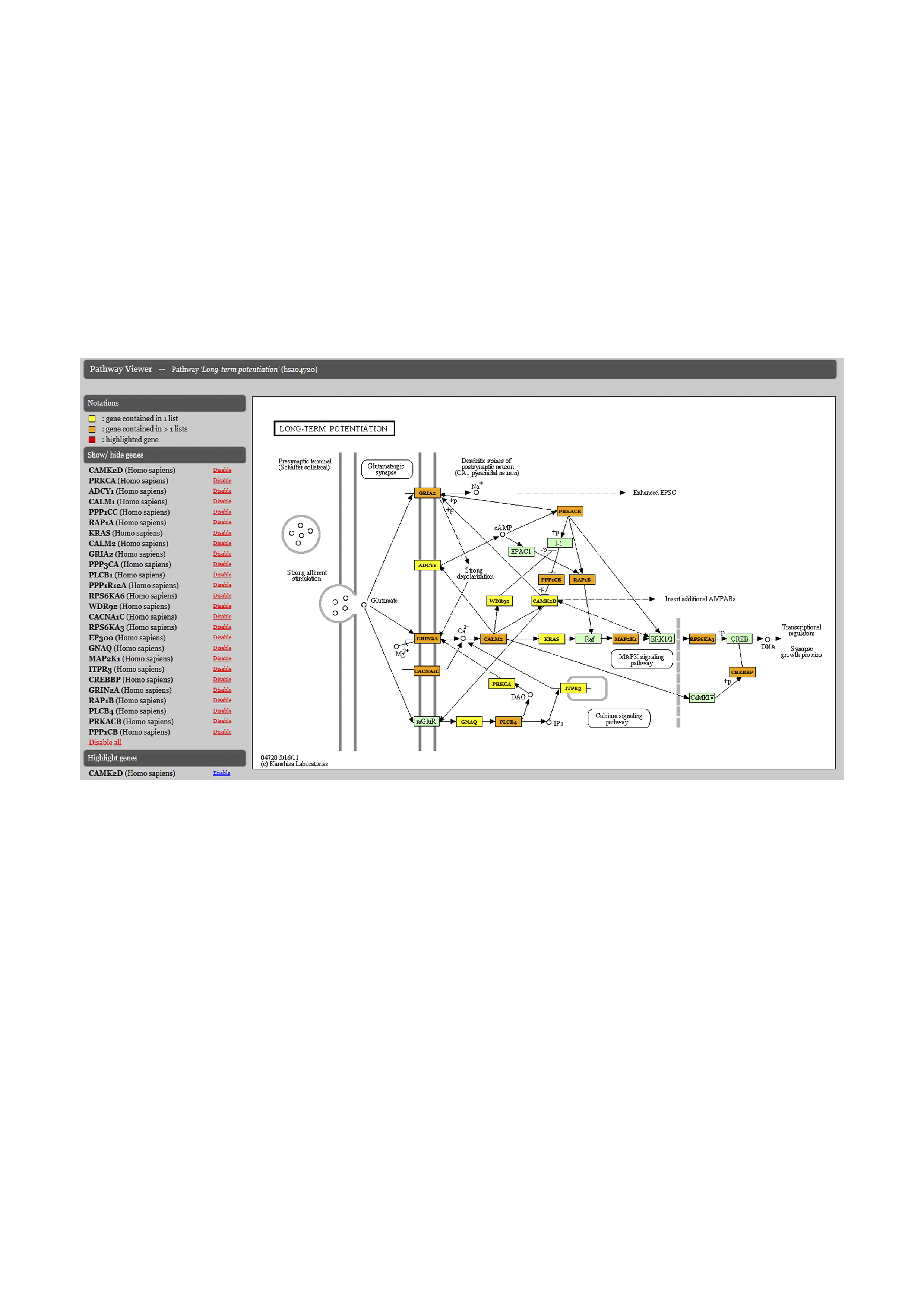

Supplement: Figure S3 — mir-154 family targets in the long term potentiation KEGG pathway. DIANA-miRPath v2.0 was used to visualize mir-154 predicted targets in the enriched long term potentiation KEGG pathway. The target prediction threshold was set at 0.85. Benjamini-Hochberg [38] correction for multiple testing controlled the P-values. (XMLNS) [file pone.0046189.s003.xml]

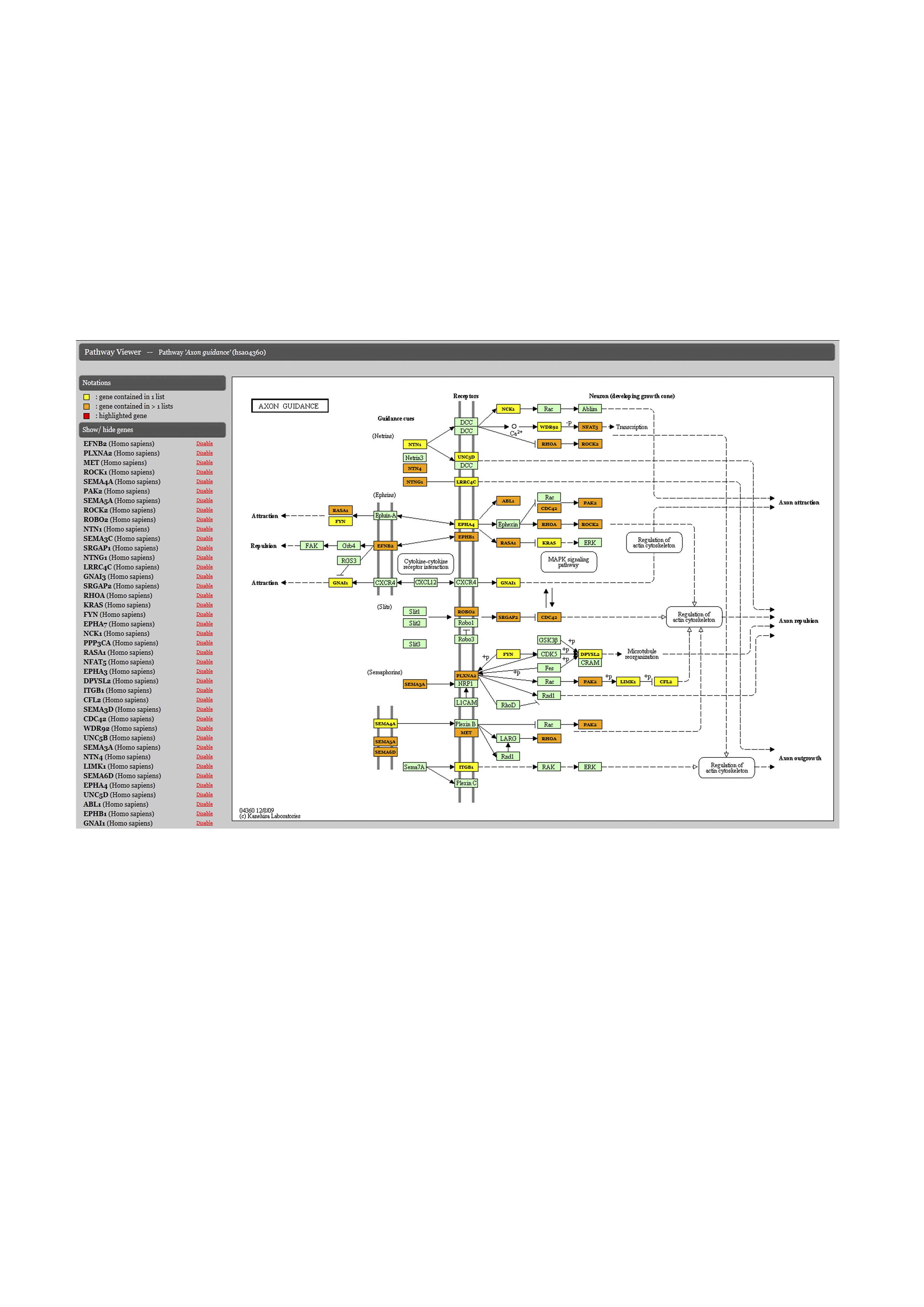

Supplement: Figure S4 — mir-154 family targets in the axon guidance KEGG pathway. DIANA-miRPath v2.0 was used to visualize mir-154 predicted targets in the enriched axon guidance KEGG pathway. The target prediction threshold was set at 0.85. Benjamini-Hochberg [38] correction for multiple testing controlled the P-values. (XMLNS) [file pone.0046189.s004.xml]

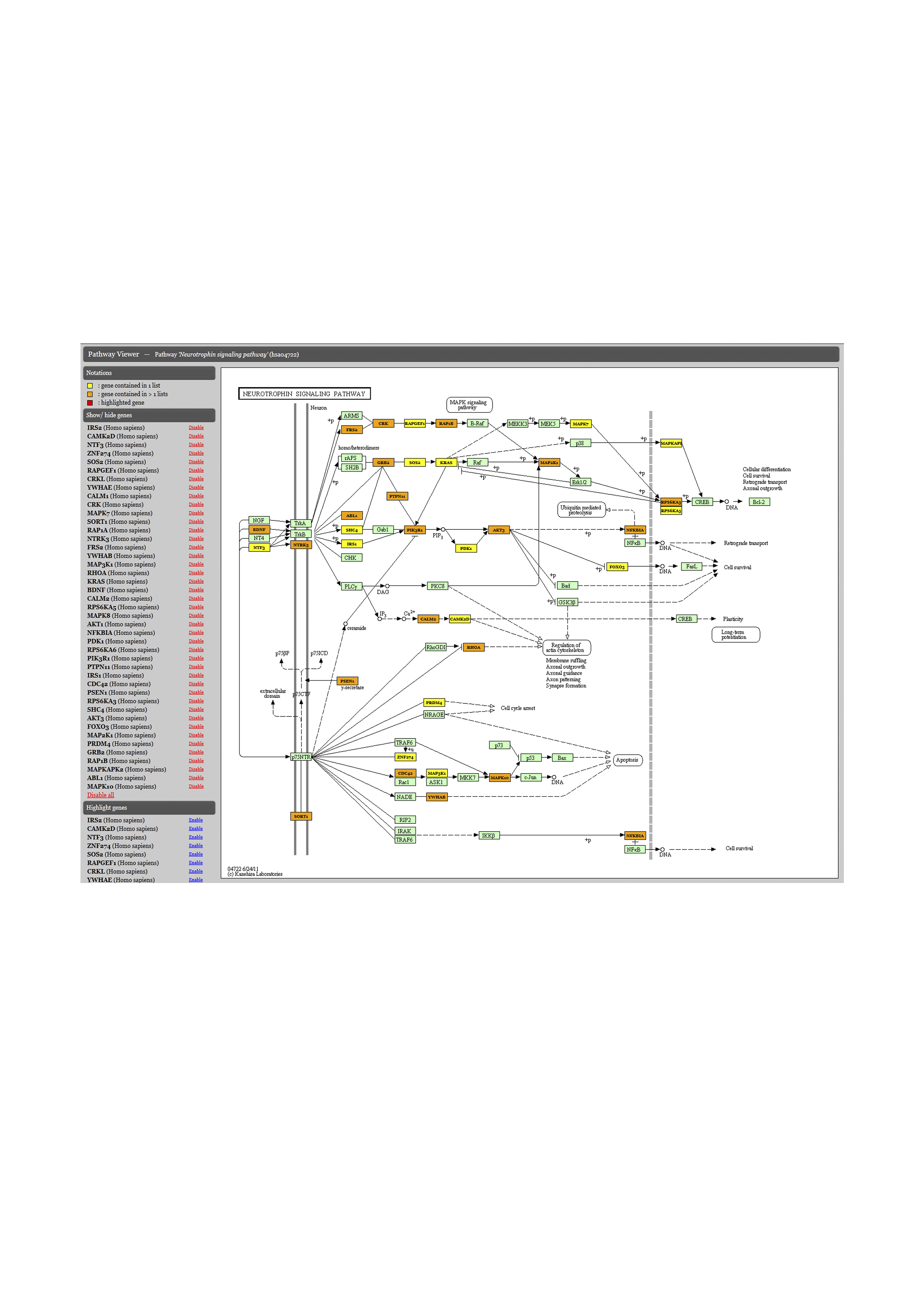

Supplement: Figure S5 — mir-154 family targets in the neurotrophin KEGG pathway. DIANA-miRPath v2.0 was used to visualize mir-154 predicted targets in the enriched neurotrophin KEGG pathway. The target prediction threshold was set at 0.85. Benjamini-Hochberg [38] correction for multiple testing controlled the P-values. (XMLNS) [file pone.0046189.s005.xml]

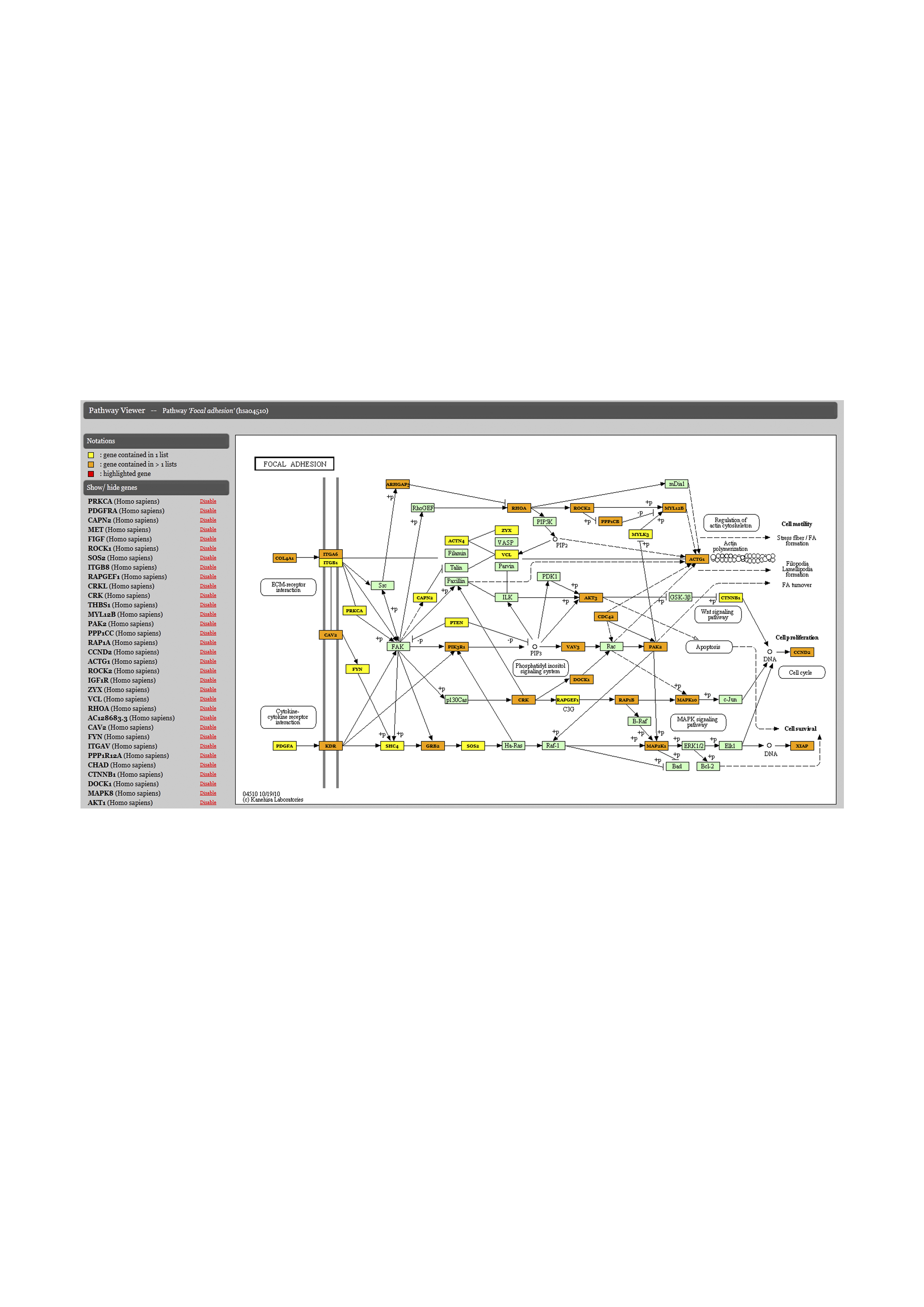

Supplement: Figure S6 — mir-154 family targets in the focal adhesion KEGG pathway. DIANA-miRPath v2.0 was used to visualize mir-154 predicted targets in the enriched focal adhesion KEGG pathway. The target prediction threshold was set at 0.85. Benjamini-Hochberg [38] correction for multiple testing controlled the P-values. (XMLNS) [file pone.0046189.s006.xml]

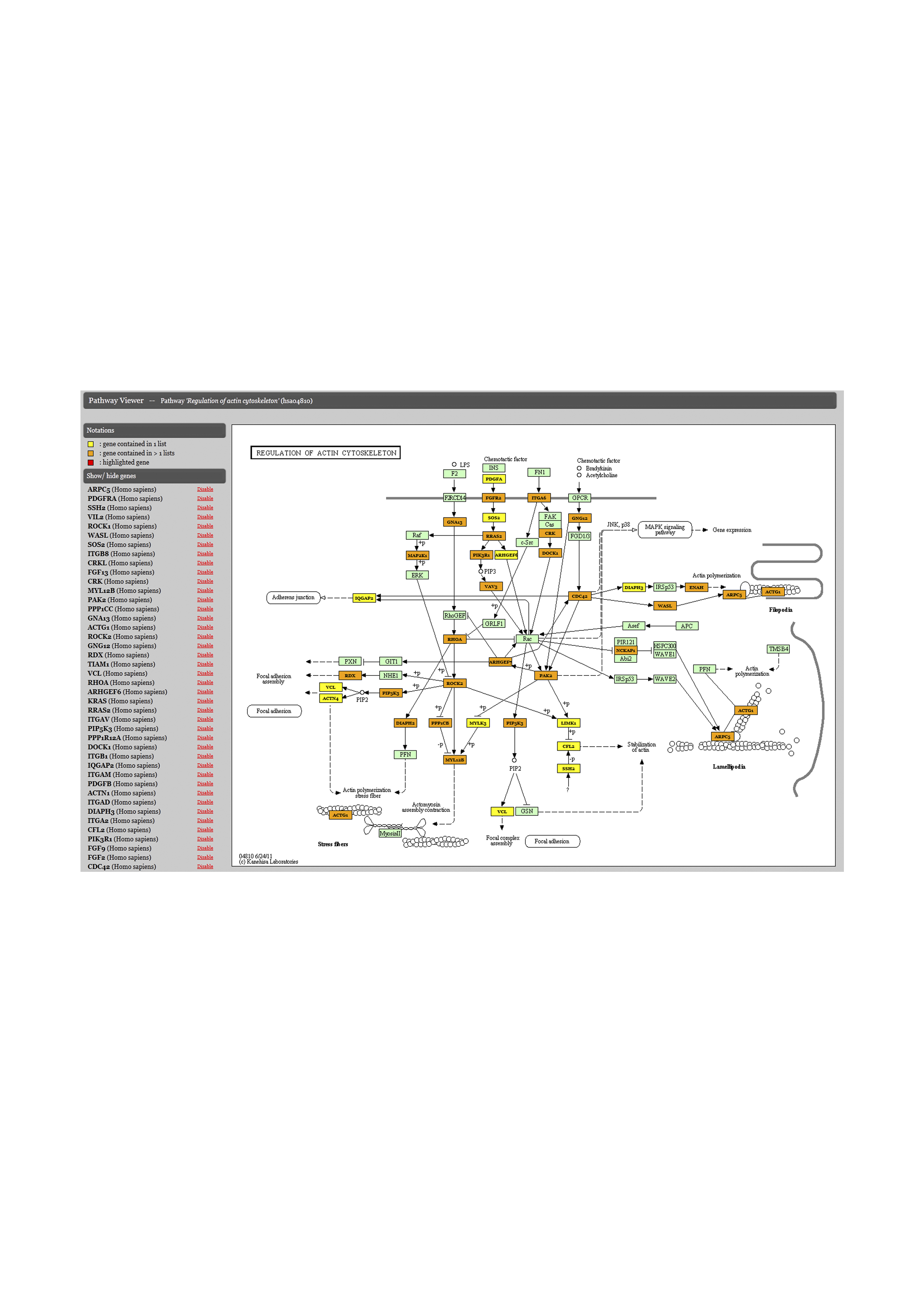

Supplement: Figure S7 — mir-154 family targets in the regulation of actin cytoskeleton KEGG pathway. DIANA-miRPath v2.0 was used to visualize mir-154 predicted targets in the enriched actin cytoskeleton KEGG pathway. The target prediction threshold was set at 0.85. Benjamini-Hochberg [38] correction for multiple testing controlled the P-values. (XMLNS) [file pone.0046189.s007.xml]
